# CDK11 Loss Induces Cell Cycle Dysfunction and Death of BRAF and NRAS Melanoma Cells

**DOI:** 10.3390/ph12020050

**Published:** 2019-04-02

**Authors:** Rehana L. Ahmed, Daniel P. Shaughnessy, Todd P. Knutson, Rachel I. Vogel, Khalil Ahmed, Betsy T. Kren, Janeen H. Trembley

**Affiliations:** 1 Department of Dermatology, University of Minnesota, Minneapolis, MN 55455, USA; ahme0056@umn.edu; 2 Masonic Cancer Center, University of Minnesota, Minneapolis, MN 55455, USA; isak0023@umn.edu (R.I.V.); ahmedk@umn.edu (K.A.); krenx@umn.edu (B.T.K.); 3 Research Service, Minneapolis VA Health Care System, Minneapolis, MN 55417, USA; shaug028@umn.edu; 4 Department of Laboratory Medicine and Pathology, University of Minnesota, Minneapolis, MN 55455, USA; knut0297@umn.edu; 5 Minnesota Supercomputing Institute, University of Minnesota, Minneapolis, MN 55455, USA; 6 Department of Obstetrics, Gynecology and Women’s Health, University of Minnesota, Minneapolis, MN 55455, USA; 7 Department of Urology, University of Minnesota, Minneapolis, MN 55455, USA

**Keywords:** CDK11, Cyclin L1, Cyclin L2, cell cycle, cancer, melanoma

## Abstract

Cyclin dependent kinase 11 (CDK11) is a protein kinase that regulates RNA transcription, pre-mRNA splicing, mitosis, and cell death. Targeting of CDK11 expression levels is effective in the experimental treatment of breast and other cancers, but these data are lacking in melanoma. To understand CDK11 function in melanoma, we evaluated protein and RNA levels of CDK11, Cyclin L1 and Cyclin L2 in benign melanocytes and BRAF- as well as NRAS-mutant melanoma cell lines. We investigated the effectiveness of reducing expression of this survival kinase using RNA interference on viability, clonal survival, and tumorsphere formation in melanoma cell lines. We examined the impact of CDK11 loss in BRAF-mutant melanoma on more than 700 genes important in cancer signaling pathways. Follow-up analysis evaluated how CDK11 loss alters cell cycle function in BRAF- and NRAS-mutant melanoma cells. We present data on CDK11, CCNL1 and CCNL2 mRNA expression in melanoma patients, including prognosis for survival. In sum, we found that CDK11 is necessary for melanoma cell survival, and a major impact of CDK11 loss in melanoma is to cause disruption of the cell cycle distribution with accumulation of G1- and loss of G2/M-phase cancer cells.

## 1. Introduction

Melanoma is the fifth most common cancer in the U.S.A., and it is the only common cancer with rapidly increasing rates and little evidence of a reversal of this trend in the foreseeable future [1,2]. While the early disease is treatable, melanoma is highly metastatic and there is a risk of metastasis even in early stage disease: 78% of melanomas are diagnosed at Stage I, yet survival for Stage I melanomas is only 84% at 10 years, and one third of melanoma deaths occur among persons initially diagnosed with the thinnest lesions (<1 mm) [1]. Surgery and chemotherapies continue to be the main strategies in the treatment of melanoma. However, there are significant side-effects from both traditional chemotherapies as well as newer therapies that target specific proteins and mutations (e.g., BRAF inhibitors). Therapies targeted to specific molecules as well as immune checkpoint blocking drugs have improved survival for melanoma. However, these therapies are not effective in all melanomas and cure rates for advanced disease remain low [3], in part because melanomas are able to develop resistance to these therapies [4,5]. Despite current treatments, only 15% of people with metastatic melanoma survive three years after diagnosis [3].

Determining and investigating cellular targets for new melanoma treatments is both urgent and crucial. Ideally, drugs directed to these targets, like cyclin-dependent kinases (CDKs), could be used on their own or in combination with other available therapies. CDKs are protein serine/threonine kinases that play key roles in the regulation of cell cycle progression as well as cellular transcription. Aberrant expression or altered activity of distinct CDK complexes results in escape of cells from cell cycle control and plays a role in malignant transformation [6,7]. There has been growing interest in Cyclin Dependent Kinase 11 (CDK11) as a target for cancer therapy [8,9]. Reducing CDK11 expression effectively blocks cell proliferation and induces cell death in breast, osteosarcoma, ovarian and liposarcoma cells and xenograft tumors [10,11,12,13,14]. However, the role of CDK11 in melanoma has not been investigated in detail.

CDK11 (formerly named PITSLRE) is essential for embryonic development and cell survival [15,16], and is well conserved throughout evolution with two almost identical CDK11 genes in humans (CDK11A and CDK11B) and one CDK11 gene in other organisms, including mice [17,18]. The predominant CDK11 protein isoforms during cell proliferation are designated p110 and p58 (CDK11^p110^, CDK11^p58^). The Cyclin L1 (CCNL1) and Cyclin L2 (CCNL2) isoforms are confirmed partner proteins for all CDK11 protein isoforms [19,20,21,22,23]. Cyclin D3 is another regulatory partner for CDK11^p58^ [24]. The CDK11^p110^ protein isoforms are detected in mammalian tissues and cell lines throughout proliferation and continue to be expressed in quiescent mouse liver [19,25]. CDK11^p110^ functions include regulation of RNA transcription and pre-mRNA splicing [19,26,27,28,29,30,31]. The CDK11^p58^ isoforms are produced via usage of an internal ribosomal entry site within CDK11 mRNA transcripts at the G2/M cell cycle transition [32]. Production of CDK11^p58^ at the G2/M transition is required for proper regulation of centriole duplication, centrosome maturation, chromatid cohesion, and spindle dynamics during mitosis [33,34,35,36,37,38].

The CDK11 p110 and p58 isoforms are cleaved by caspases to generate a 46–50 kDa protein that contains the catalytic portion of the protein as well as a p60 N-terminal protein [39,40,41,42,43]. During apoptosis, CDK11^p60^ re-distributes from the nucleus to the mitochondria [44]. Ectopic expression of CDK11^p60^ can partially disrupt the mitochondrial membrane potential, cause cytochrome *c* release, and induce apoptosis [44].

To understand the role of CDK11 in melanoma, we evaluated levels of CDK11 in benign melanocytes and melanoma cell lines. Next, we investigated the effects of CDK11 downregulation on melanoma cell viability, clonal survival and tumorsphere formation as well as on various signaling pathways and cell cycle distribution. Our data presented herein demonstrate that CDK11 is highly expressed in both BRAF- and NRAS-mutated melanoma cell lines. Loss of CDK11 induces cell cycle dysfunction and death of BRAF- and NRAS-mutant melanoma cell lines. Overall, our data indicate the dependence of melanoma cells on CDK11 expression for survival.

## 2. Results

### 2.1. CDK11A and CDK11B mRNA Expression in Non-Transformed Melanocytes and Melanoma Cell Lines

We determined steady state mRNA expression levels for both CDK11 genes in cultured cells, comparing several BRAF- and NRAS-mutant melanoma cell lines and using adult primary human epidermal melanocytes as a reference control (Table 1). Data from quantitative real-time reverse transcriptase PCR (qRT-PCR) are summarized in Table 2. CDK11 mRNA levels were lower in malignant cells compared to primary melanocytes in all of the melanoma cell lines tested, except for CDK11A mRNA in WM39 cells. We include the data for MYC as an example for a gene generally showing higher mRNA expression levels in melanoma cells relative to non-transformed melanocytes.

### 2.2. Expression of CDK11 Protein Complex Constituents in Primary Melanocytes and Melanoma Cell Lines

**Figure 1:**
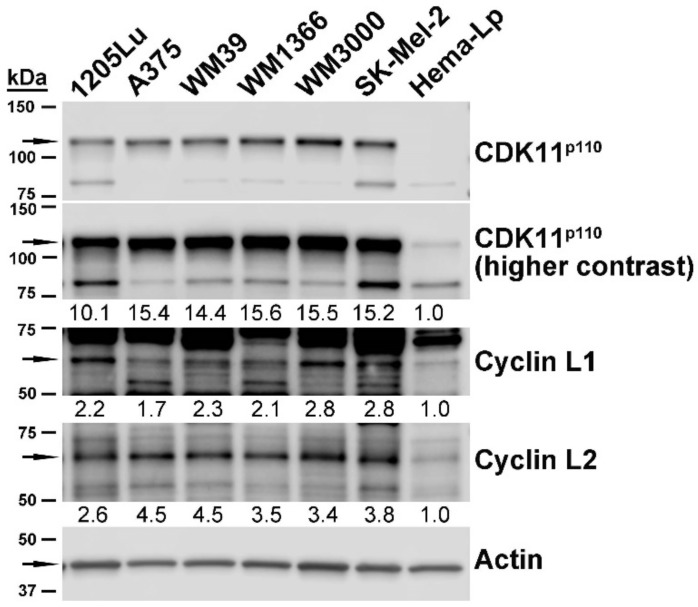
Expression of CDK11 protein complex members in untransformed and malignant melanocytes. Immunoblot analysis of cultured melanocyte cell lines, as indicated above the blots. Proteins detected are indicated on the right side of the blots. Arrows on the left side of blots indicate dominant protein isoform bands. Actin signal was used as the loading control. Quantitation of signal relative to actin is indicated below each protein band.

We examined the steady-state protein expression levels for CDK11, Cyclin L1α and Cyclin L2α in melanoma and primary melanocyte cells. CDK11^p110^ and partner cyclins L1α and L2α were highly expressed in melanoma cells relative to non-transformed melanocytes (Figure 1). The CDK11^p110^ isoforms were detected at levels averaging 14-fold higher than primary melanocytes. Cyclin L1α and Cyclin L2α proteins were detected at levels 2 to 4-fold higher than in the non-malignant cells.

Protein expression levels for the CDK11 complex members do not mirror the corresponding mRNA levels in these melanoma cell lines. Further, although a C-terminal antibody was used that is capable of recognizing CDK11^p58^, this mitosis-specific isoform was not detected in asynchronously growing melanoma cells.

### 2.3. Down-Regulation of CDK11 Using siRNA Decreases BRAF- and NRAS-Mutant Melanoma Cell Viability

**Figure 2:**
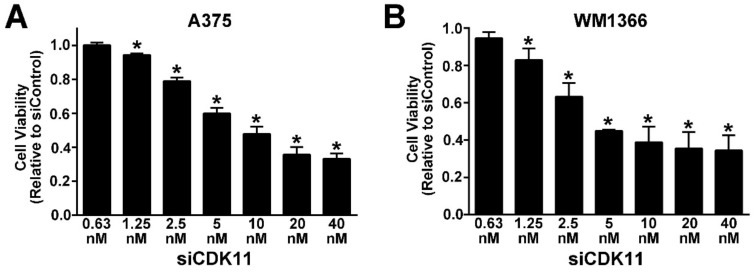
siRNA-mediated down-regulation of CDK11 in BRAF- and NRAS-mutant melanoma cells decreases cell viability. A375 (**A**) and WM1366 (**B**) cells were transfected with increasing concentrations of siRNA directed against CDK11. After 96 h, cell viability was determined relative to the siControl transfected cells. Means ± SE from three experiments are presented. * = *p* < 0.05.

Using a siRNA sequence previously validated for both CDK11 gene transcripts in other cancer types, we evaluated the effects of CDK11 down-regulation on melanoma cell viability [10,34]. Cell viability in BRAF-mutant A375 and NRAS-mutant WM1366 cells was determined after transfection of siCDK11 or siControl siRNAs, using a concentration range from 0.63 nM to 40 nM. Cell viability in both cell lines, as determined by a mitochondria-based MTS-related assay, showed little change at 48 h of CDK11 down-regulation. A loss of up to approximately 35% viability was observed at 72 h post-transfection (data not shown). At 96 h post-transfection, reduced viability was observed with 1.25 nM siCDK11, and increasing effect was noted in a linear manner through 20 nM siCDK11 (Figure 2).

### 2.4. Loss of CDK11 Expression Has a Negative Impact on the Ability of Melanoma Cells to Form Colonies and Tumorspheres

**Figure 3:**
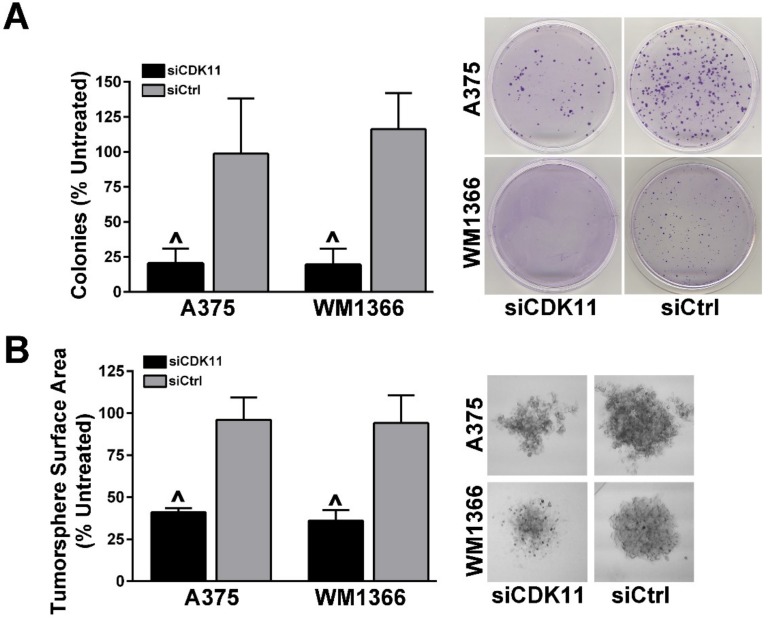
Down-regulation of CDK11 inhibits clonal survival and tumorsphere formation in melanoma cells. A375 and WM1366 cells were transfected with 30 nM siRNAs as indicated in the legends and as described in materials and methods. (**A**) For clonal survival analysis, cells were plated onto 35 mm plates 48 h post-transfection and colonies were stained and counted seven days after plating. Left: The chart presents means ± SD from three experiments with three replicate plates each. ^ = *p* < 0.0001. Right: Representative crystal violet stained colonies on 35 mm plates. Cell lines are indicated to the left of images and siRNA transfections are indicated below plate images. (**B**) For tumorsphere formation, cells were plated into 96-well ultra-low attaching plates 48 h post-transfection and images captured 96 h after plating. Left: The chart presents means ± SD from three experiments with three areas each. ^ = *p* < 0.0001. Right: Representative tumorsphere images. Cell lines are indicated to the left of images and siRNA transfections are indicated below plate images.

We used a clonal survival assay in A375 and WM1366 cells, each transfected one time with 30 nM siCDK11 or siControl siRNAs or left untreated. Forty-eight h after transfection, the cells were collected, counted and plated in triplicate into 35 mm plates. After 7 days of incubation, the cell colonies were stained with crystal violet and counted. Down-regulation of CDK11 protein expression resulted in a more than 75% reduction in colony formation compared to either siControl treated or untreated cells in both BRAF- and NRAS-mutant cell lines (Figure 3A).

We next employed tumorsphere formation assays in A375 and WM1366 cells. Cells were transfected in the same manner as the clonal survival assays. Forty-eight h after transfection, cells were collected, counted, and plated in triplicate into ultra-low attachment plates. After 96 h, images of the tumorspheres were captured and measured. Transfection of A375 and WM1366 cells with control siRNAs resulted in the formation of robust tumorspheres that were comparable to tumorsphere size and morphology in untreated cells. Downregulation of CDK11 caused much smaller tumorspheres to form, which were also less dense and loosely formed (Figure 3B).

### 2.5. Effects of CDK11 Signaling Reduction on Melanoma Growth Pathways

We examined the effects of CDK11 siRNA-mediated knockdown on some melanoma growth and signaling pathways. Although CDK11 knockdown was robust (Table 3), downstream changes in these pathways at 48 and 72 h post-transfection were generally modest with a few exceptions. Dramatic loss of cMYC protein was observed in both A375 and WM1366 cells. Loss of CDK11 in NRAS-mutant WM1366 melanoma moderately downregulated β-catenin expression and activation; conversely, β-catenin protein expression increased after siCDK11 transfection in A375 and WM39 cells (Table 3 and data not shown). Seventy-two h post-transfection, A375 showed reduction in Survivin expression and WM1366 showed reduction in MCL-1 expression.

### 2.6. CDK11 Impact on Pan-Cancer Gene Expression

We observed strong effects of CDK11 loss on melanoma viability, clonal survival, and tumorsphere formation 96 h or longer after CDK11 downregulation; however, relatively modest effects of CDK11 loss on classical melanoma growth pathways were seen at the protein expression level. To identify early events occurring in melanoma cells due to blocking CDK11 expression, we evaluated more global changes in gene expression using the PanCancer Pathways Panel (NanoString Technologies, Seattle, WA, USA).

**Figure 4:**
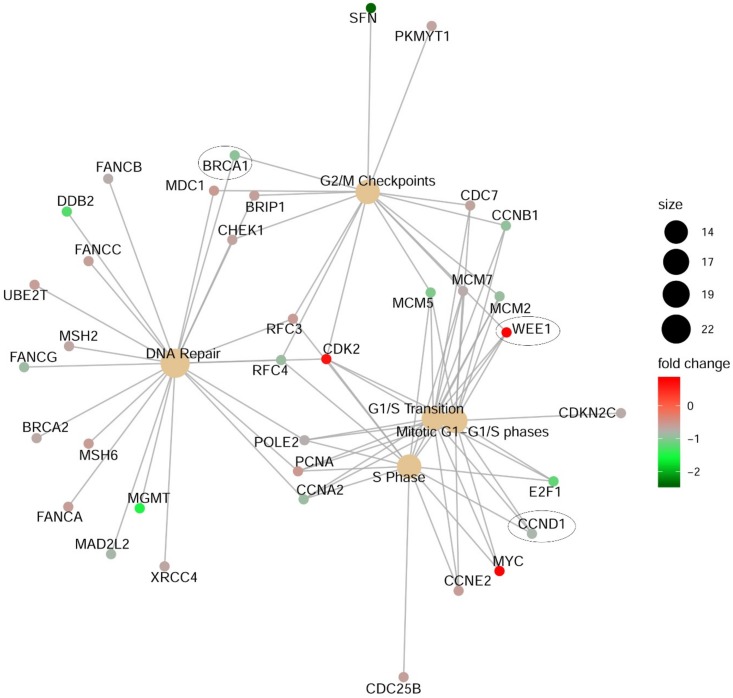
Signaling pathway nodes effected by loss of CDK11 in A375 melanoma cells following PanCancer Reactome pathways analysis. A375 cells were transfected with siRNA directed against CDK11 or control siRNA. After 48 h, cells were collected for RNA purification. Gene names with an ellipse drawn around them represent genes that were similarly altered in both A375 and WM1366 cells after q-RT-PCR verification. Fold change in gene expression is indicated by the red (increased) to green (decreased) scale shown. Node circle size represents the number of genes altered in that pathway.

This panel allows gene expression analysis of 770 genes from 13 cancer-associated pathways using an amplification-free single molecule counting technology. A375 cells were transfected with siCDK11 or siControl, and the RNA purified for analysis at 48 h post-transfection (*n* = 3 per condition). Significant differential expression due to decreased CDK11 expression was observed in 97 genes: 24 genes were upregulated and 73 genes were downregulated. The results for all statistically significant differentially expressed genes are summarized in Table S1. A Reactome pathway enrichment analysis was also performed. The results indicated that DNA repair and several cell cycle-related phases, transitions and checkpoints were key nodes effected by loss of CDK11 expression. Observed and predicted genes identified and their relationship to DNA repair and cell cycle events are depicted in Figure 4.

In accordance with the observed increase in β-catenin protein expression in BRAF-mutant cells (Table 3), mRNA levels for β-catenin were also increased in the PanCancer panel after siCDK11 treatment (1.5-fold; *q*-value = 0.004). MAPK1, the gene for protein Erk 2, was down-regulated 0.67 fold (*q*-value = 4.68 × 10^−5^) on the PanCancer panel, and the protein level was decreased 0.87 fold at 48 h as well (Table 3). In contrast, MYC mRNA level showed significant increase at 48 h post-siCDK11 transfection in A375 cells (1.72-fold; *q*-value = 0.001), whereas the protein levels decreased at 48 h and especially at 72 h (Table 3 and Table 4).

### 2.7. CDK11 Loss Rapidly Disrupts Cell Cycle Function in Melanoma

Results from the PanCancer analysis strongly indicate that disruption of cell cycle checkpoints are a major and early event upon CDK11 downregulation. First, we evaluated several of the PanCancer results by q-RT-PCR in A375 cells. As shown in Table 4, the changes observed for eight cell cycle-associated genes by the PanCancer panel were confirmed by q-RT-PCR at 48 h post CDK11 knockdown. We also examined expression of these same genes in WM1366 cells and found that some of the changes observed at the transcript level in A375 cells were duplicated in the NRAS-mutant cell line. Specifically, CCNB1, CCND1, WEE1, and BRCA1 showed expression level changes in the same direction for WM1366 as compared to A375 cells.

**Figure 5:**
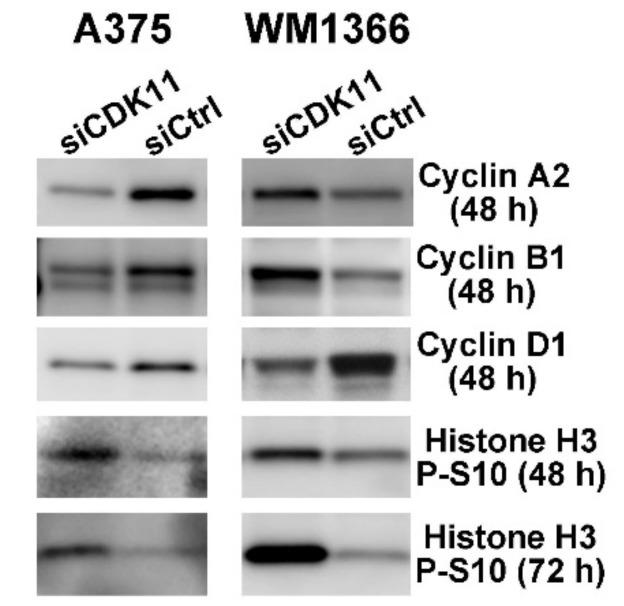
Immunoblot analyses for cell cycle-related proteins following siRNA-mediated down-regulation of CDK11 in melanoma cells. Immunoblot analysis of A375 and WM1366 cell lysates following 30 nM siRNA transfection. SiRNAs transfected are indicated above the blots, proteins detected and time point are indicated on the right side of the blots. CDK11^p110^ knockdown verification is indicated in Table 5. Actin signal was used as the loading control.

We also investigated the impact of CDK11 downregulation on cyclin genes by examining changes in their protein levels at 48 and 72 h in both A375 and WM1366 cells. The results shown in Figure 5 and Table 5 demonstrate that the CCND1 RNA changes in each cell line translated to a similar change in Cyclin D1 protein level. Loss of Cyclin D1 protein was also observed after CDK11 down-regulation in SK-Mel-2 and WM39 cells (data not shown).

For A375 cells, the same concordance between mRNA and protein levels is true for Cyclin B1 and Cyclin A2. In WM1366 cells, in contrast to the mRNA levels, Cyclin A2 and Cyclin B1 protein levels increased slightly at 48 h and more dramatically at 72 h. Cyclin E2 protein was not detected in either cell line.

Given the role for CDK11 in mitotic regulation and the increased levels of Ser10 phosphorylation on Histone H3 in mitosis, we examined the impact of CDK11 loss on Histone H3 phospho-Ser10 levels. In both A375 and WM1366 cell lines, the amount of phospho-Ser10 Histone H3 detected was higher at 48 h post-siCDK11 transfections than control cells, and the levels of phospho-Ser10 Histone H3 continued to increase at 72 h (Figure 5, Table 5, and data not shown).

**Figure 6:**
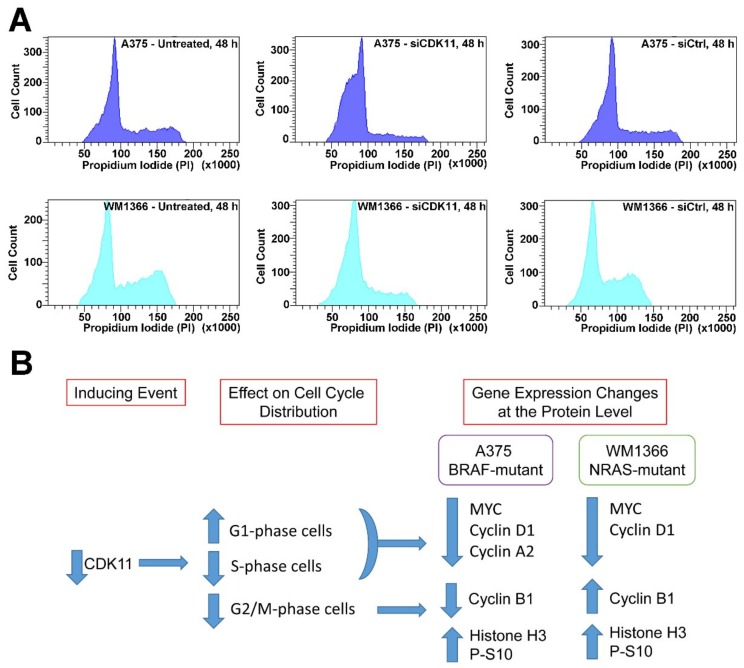
FACS analysis for cell cycle composition following siRNA-mediated down-regulation of CDK11 in melanoma cells and summary of cell cycle-related results. (**A**) PI-based FACS analysis for DNA content in untreated and siCDK11 and siControl transfected A375 (upper panels) and WM1366 (lower panels) cells is shown. The identity of the treatment type and time point is indicated within each panel. (**B**) A cartoon summary of the cell cycle-based alterations in melanoma cells after CDK11 downregulation and the associated changes in protein expression levels.

To further elucidate CDK11 control of cell cycle in melanoma cells, we performed FACS analysis of A375 and WM1366 cells. At 48 h of siRNA-mediated CDK11 knockdown, both A375 and WM1366 demonstrated a strong accumulation of cells into G1 phase and loss of cells from G2/M phases (Figure 6A and Table 6). We summarized the results of our investigations into how CDK11 loss alters cell cycle distribution and function in BRAF- and NRAS-mutant melanoma in Figure 6B.

### 2.8. CDK11, CCNL1 and CCNL2 Are Co-Expressed in Human Melanoma Patient Samples

Gene mutation is not a major component of CDK11A, CDK11B, CCNL1 or CCNL2 function in melanoma. Information from human melanoma patient data derived from The Cancer Genome Atlas (TCGA) indicated that 5% or less of the samples contain a mutation in each of these genes, and the majority of mutations detected were missense in origin with unknown significance [45,46]. Like the CDK11 genes, the CCNL2 gene is located on chromosome 1p, and the genomic data showed that gene amplification in three patients and deep gene deletion in four patient samples all occurred simultaneously for these three genes. We also examined CDK11, CCNL1 and CCNL2 gene expression levels using the TCGA RNA-seq data. Using a z-score cut-off of 1.5, alteration of mRNA levels (up- or down-regulation, not mutation) was observed in 17% of samples for CDK11A, 19% for CDK11B, 9% for CCNL1, and 10% for CCNL2. These alterations were described as a tendency toward co-occurrence with significance (*q*-value ≤ 0.001) for the pairings of: CDK11A + CDK11B; CCNL1 + CCNL2, CDK11A + CCNL1, and CDK11A + CCNL2 [45,46]. Thus, altered expression of these genetic partners can occur within the same tumor.

**Figure 7:**
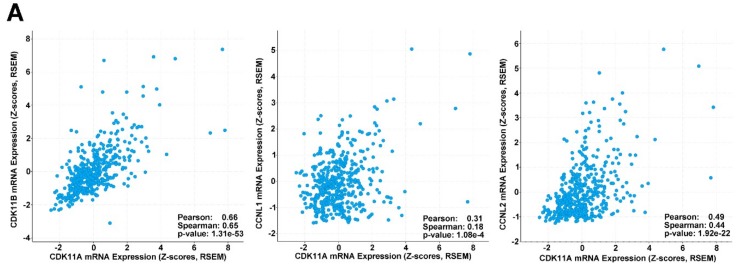

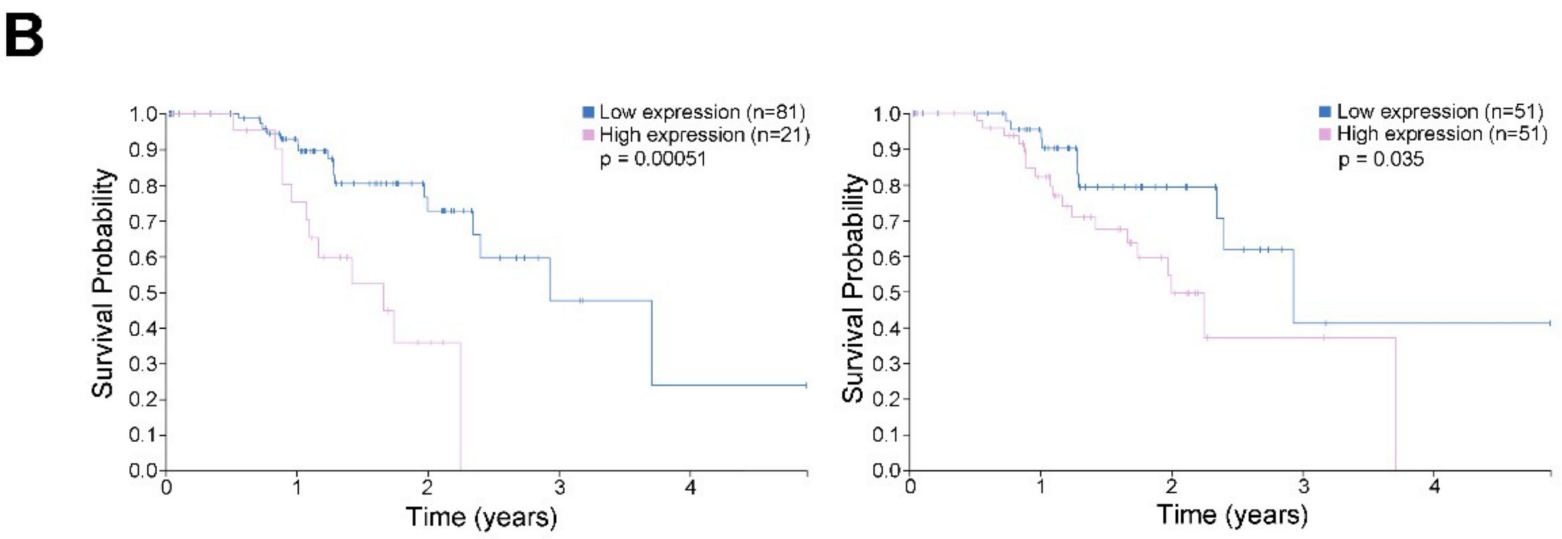
Expression of CDK11 and associated genes in melanoma patient samples and association with survival. (**A**) Analysis of co-expression of CDK11A, CDK11B, CCNL1 and CCNL2 mRNA levels in melanoma patient samples from The Cancer Genome Atlas (Pan-Cancer Atlas; *n* = 443 samples). Correlation analysis and *p*-values provided within each panel. Analysis performed using cBioPortal. RSEM, RNA-Seq by Expectation–Maximization. (**B**) Survival analysis in melanoma patients indicating that high CDK11A mRNA expression is unfavorable. Kaplan-Meier plots are shown for best expression cut-off (1.82; Left panel) and median expression cut-off (1.38; Right panel). *n* = 102 human cutaneous melanoma patient samples. *p*-values for log-rank tests are provided within each panel. Analysis performed by Human Protein Atlas using data from The Cancer Genome Atlas. Image credit: Human Protein Atlas.

Correlation of gene expression levels was also examined in TCGA RNA-seq data. There were significant positively co-expressed mRNA levels for CDK11A and CDK11B and for CDK11A and CCNL2 (Figure 7A). CDK11A and CCNL1 mRNA levels correlated to a lesser extent (Figure 7A), as was observed for CDK11B and CCNL2 (data not shown).

### 2.9. High CDK11A mRNA Expression is Associated with Decreased Patient Survival

Pathological analysis performed by the Human Protein Atlas for CDK11A in melanoma patients (derived from TCGA) indicates that high CDK11A mRNA expression is an unfavorable prognostic marker [47]. Survival curves for CDK11A in melanoma patients are show in Figure 7B. The RNA-seq data used in this survival analysis derived from melanoma tumors representing primary sites. A summary of the characteristics of the 102 patients included in the survival curves presented is found in Table S2.

## 3. Discussion

CDK11 represents an important therapeutic target for many cancers, and the results we report here demonstrate for the first time a dependence of melanoma cells on high CDK11 protein expression levels. By acutely down-regulating CDK11 protein expression via siRNA transfection, both BRAF- and NRAS-mutant melanoma cells showed significantly reduced cell survival and disrupted cell cycle function. Blocking CDK11 function has potential impact on the survival of both mitotically cycling and non-cycling malignant cells, because the expression and functions of CDK11 isoforms affect both proliferating and quiescent cells. Development of a CDK11-based cancer cell-specific treatment strategy would bring together current concepts of targeting cell cycle control CDKs with that of targeting essential transcriptional control CDKs [7,48,49]. Additionally, as is true for many cancer types, MYC expression is related to aggressive forms of melanoma and is being investigated as a potential therapy target in melanoma. Treatment-induced loss of CDK11 has the beneficial effect of also reducing cMYC protein levels.

Investigation of chromosome 1p deletions in melanoma demonstrated abnormalities in the CDK11 genes (previously designated as CDC2L1 and CDC2L2) [50]. One allele of the CDK11 gene complex was found to be deleted in six of 14 melanoma cell lines; however, most cell lines with CDK11 gene loss as a result of chromosome 1p deletion also contained two other copies of chromosome 1 with intact CDK11 genes. These data suggest a possible theory in which stably reduced CDK11 expression levels, due to chromosome 1p loss sometime during the oncogenic process, represents a chronic but not death-inducing state within cells with the potential to promote malignancy. In subsequent work, mice were generated in the Nelson laboratory which expressed only 1 allele of CDK11 to examine the effects of reduced CDK11 expression in skin [51]. These mice showed reduced CDK11 expression in skin tissue. Following a skin carcinogenesis regimen with 7,12-dimethylbenz[α]anthracene (DMBA) and 12-*O*-tetradecanoylphorbol-13-acetate (TPA), the average number of skin papillomas per mouse was 3-fold higher in the single copy CDK11 gene mice compared to mice with two copies of CDK11. In contrast to CDK11 down-regulation in melanoma or other cancer cells with pre-existing dependency on CDK11 expression, chronic and moderate reduction in CDK11 expression over time in pre-malignant cells could contribute to decreased cell death due to reduced levels of the CDK11 p46 and p60 cleavage products as well as dysregulation of normal transcriptional and cell cycle control programs, thus acting to promote malignant characteristics and survival of cells.

Lower expression levels of CDK11^p110^ protein in several melanoma cell lines as well as melanoma patient samples relative to primary human foreskin-derived melanocytes were reported [50]. In this publication, CDK11 protein levels in A375 cells were determined to be higher than in the normal melanocytes [50], similar to the results we present here. By contrast, our data show that CDK11^p110^ protein levels were consistently higher in all examined melanoma cell lines relative to adult skin-derived primary epidermal melanocytes. It should be noted that not only are the melanoma cell lines distinct in these two analyses, with the exception of A375, but also different benign melanocytes were used for comparison. Several phosphorylation sites and regions are reported for CDK11, and the CDK11 kinase domain contains the classic CDK activation sites [17,30,52,53]. However, there is limited knowledge as to how CDK11 phosphorylation status influences CDK11 function and cell viability, especially with respect to melanoma.

We have demonstrated that one of the mechanisms by which blocking CDK11 expression causes melanoma cell death is by disrupting cell cycle progression. We observed altered G1 and G2/M cyclin gene expression as well as accumulation of phospho-Ser 10 Histone H3. Histone H3 phosphorylation at Ser10 is associated both with condensation of mitotic chromosomes producing a transcriptionally repressed state as well as with signal induced gene activation during interphase [54,55]. Histone H3 Ser10 phosphorylation during mitosis is at a much higher density compared to that in interphase. Complete knock-out of CDK11 in mouse has previously been shown to associate with mitotic arrest and accumulation of phosphorylated Histone H3 [15]. Here we have shown that knockdown of CDK11^p110^ protein levels by more than 50% caused accumulation of phospho-Ser10 Histone H3 at 48 h, with continued accumulation at 72 h post-transfection.

We also observed increased G1-phase cells after CDK11 knockdown, similar to what was reported in breast cancer, another epithelial cancer [13]. In contrast, CDK11 knockdown in esophageal squamous cell carcinoma cells induced G2/M accumulation [56]. CDK11 and Cyclin L1 were recently reported to regulate the cell cycle in non-transformed *Bombyx mori* (silkworm) cells. Manipulation of CDK11 and Cyclin L1 expression levels in silkworm cells had two opposing effects. Overexpression of CDK11 and/or Cyclin L1 in non-cancer silkworm cells resulted in an increased population of G1 phase cells and a corresponding decreased G2/M phase population. Conversely, blocking CDK11 and/or Cyclin L1 expression using siRNAs arrested these cells in G2/M, with decreased G1 phase cells. Both overexpression and inhibited expression of CDK11 and/or Cyclin L1 reduced the viability of these cells, suggesting a window of appropriate CDK11 levels in these non-cancer cells [57]. The effects of CDK11 loss in silkworm cells align with similar effects during mouse embryo development [15].

Even though both BRAF-mutant A375 and NRAS-mutant WM1366 cells responded similarly to CDK11 down-regulation with respect to loss of cell survival capability and accumulation of G1-phase cells, these 2 types of melanoma did not respond identically at the molecular level. G1 and G2/M cyclin changes were different with the exception of a consistent loss of Cyclin D1. Further, both cell lines showed increased WEE1 mRNA, decreased BRCA1 mRNA, and loss of c-MYC protein. A375 cells were unique in a dramatic loss of SFN transcript, which encodes 14-3-3 protein sigma. 14-3-3 family proteins recognize and bind Histone H3 phosphorylated at Ser10, acting as “readers” of the histone code and as a platform for assembly of transcriptional regulatory factors, participating in cell cycle control and RAF kinase regulation among other functions [58]. Many 14-3-3 isoforms interact with CDK11^p110^, and these interactions peak during G2/M in synchronized A375 cells [59]. It has also been shown that loss of 14-3-3 protein sigma affects proper regulation of interphase versus mitotic protein translation [60]. Human cancer cells depleted of 14-3-3 protein sigma do not suppress cap-dependent or stimulate cap-independent translation, events needed at the mitosis transition, resulting in reduced mitotic-specific expression of CDK11^p58^, impaired cytokinesis, and accumulation of binucleate cells. It is possible that downregulation of SFN is contributing to blockade of the transcriptional program and accumulation of G1 phase cells.

It is not surprising that the genes CDK11A and CDK11B and CCNL2, which are all located on chromosome 1p, showed good correlation of expression at the mRNA level in melanoma patient tumor tissues. There was weaker but still significant correlation between CDK11A and CCNL1 transcript levels as well. Patient survival data from TCGA suggest that high level expression of CDK11 mRNA is a marker of poor prognosis in melanoma. These data agree with our contention that melanoma cells are dependent on robust CDK11 protein expression for survival. Higher CDK11 protein expression levels are associated with poorer patient survival in osteosarcoma and ovarian cancer [11,14]. The association of CDK11 levels with patient survival in breast cancer is not yet clear. In one publication, elevated CDK11 protein expression in breast cancer tissues was associated with poor differentiation of tumor for breast cancer patients, but statistical significance was not achieved for survival at 72 months of follow-up [13]. Consistently, experimental down-regulation of CDK11 in various breast cancer cell lines definitively resulted in cell death [10,13].

In summary, we found elevated levels of CDK11 protein expression in both BRAF- and NRAS-mutant melanoma cell lines compared to benign melanocytes. We present the novel finding that down-regulation of CDK11 protein expression reduced cell survival and disrupted cell cycle function in both BRAF- and NRAS-mutant melanomas. Targeting CDK11 has potentially important clinical relevance for the development of a novel therapy that spans multiple melanoma sub-types. Early CDK inhibitors were non-specific and had significant side-effects while not being very effective. Mechanisms of resistance with the non-specific CDK inhibitors have also been demonstrated [61]. Based on our findings there may be promise in specific targeting of CDK11 for melanoma, as previously demonstrated using malignant cell-specific CDK11 downregulation in breast cancer [10]. The work presented in this report is early and should be expanded in additional melanoma cell lines and in animal models. Thus, our novel findings warrant future work to understand CDK11 in melanoma at both the cellular and in vivo levels.

## 4. Materials and Methods

### 4.1. Cell Lines and Culture

SK-Mel-2 cells were obtained from ATCC (Manassas, VA, USA), and grown according to ATCC recommendations with 1% Pen/Strep. WM1366 and WM39 cells were obtained from Coriell Cell Repositories (Camden, NJ, USA). WM39 cells were grown according to Coriell Cell Repositories recommendations. WM1366 cells were grown in RPMI-1640 (SH30255.01 HyClone Laboratories, Logan, UT, USA) with 10% fetal bovine serum and 1% Pen/Strep. A375 cells were a kind gift from Dr. James McCarthy (University of Minnesota, Minneapolis, MN, USA); these cells were authenticated by IDEXX BioResearch (Columbia, MO, USA) using a nine marker STR profile. A375 growth conditions were DMEM high glucose (HyClone SH30243.01), 10% FBS, 1% Pen/Strep. All cell lines were grown in an incubator at 37 °C with 5% CO_2_. All cells had undetectable levels of mycoplasma when thawed, and were maintained in culture for up to 2 months.

### 4.2. Oligonucleotides

Standard chemistry siRNAs were obtained from Dharmacon (Lafayette, CO, USA). The sense strand sequence for siCDK11: 5’-gagcgagcagcagcgugugdTdT-3’ [34]. The control siRNA (siControl) used was siNon-targeting #2 (D-001810-02, Dharmacon).

### 4.3. Cell Viability Assays

Reverse transfection of A375 and WM1366 cells was performed. Transfection complexes were formed by serial dilution (2-fold dilution series) of 20 µM siRNA into a combination of Dharmafect 1 and OPTI-MEM resulting in siRNA concentrations ranging from 40 nM to 0.625 nM. A master 96-well plate was loaded with 125 µL/well for each concentration, then 25 µL/well was transferred to each of three Primaria plates. Cells were trypsinized and plated (3000 cells/well for WM1366; 2,500 cells/well for A375) in 100 µL of complete 5% FBS antibiotic-free media directly onto the transfection mixture. After 24 h of incubation at 37 °C/5% CO_2_, an additional 140 µL of complete 5% FBS media was added to all wells in all plates. At 48, 72, and 96 h following transfection initiation, CellTiter 96^®^ Aqueous One Assays were performed according to the manufacturer’s instructions (Promega Corp., Fitchburg, WI, USA). Absorbance values for media alone were subtracted from the experimental values.

### 4.4. Clonal Survival Assays

SiRNA transfection complexes were formed by combining 30 nM siRNA with 10 µL Dharmafect 1 and OPTI-MEM in total volume of 400 µL. After a 20 min incubation, 1.6 mL of antibiotic-free media containing 5% FBS was mixed in and the total volume was added to cells at 50% confluence on 60 mm plates after the previous growth media was removed. After 5 h of incubation at 37 °C/5% CO_2_, an additional 2 mL of complete media was added to each plate. At 48 h post-transfection, the cells were collected using trypsin, counted, and plated in triplicate at a concentration of 500 cells per 35 mm plate in complete media with 5% FBS. The media was replaced after 4 days. Seven days after plating, the cells were stained with crystal violet for 20 min (1X PBS containing 1% (*v*/*v*) methanol, 1% (*v*/*v*) formaldehyde and 0.05% (*w*/*v*) crystal violet); the stain was then removed and plates were washed by immersion in water with continuous water flow. Plates were air-dried, colonies were counted using the OpenCFU program, and plates were scanned.

### 4.5. Tumorsphere Formation Assays

Cells were transfected in the same manner as the clonal survival assays. At 48 h after transfection, cells were collected using trypsin, counted, and plated in a final volume of 200 µL/well of complete media with 10% FBS in a 96-well Ultra Low Attachment plate (1500 cells/well for WM1366; 600 cells/well for A375; Corning, Tewsbury, MA, USA). The plate was spun in a centrifuge with a plate rotor for 5 min at 200× g. At 96 h after plating, pictures were captured of the different conditions with transmitted light microscopy (Moticam X camera, Motic, Richmond, BC, Canada and an Axiovert microscope on the 5× objective, Zeiss, Thornwood, NY, USA).

### 4.6. Immunoblot Analysis

After cell pellets from cultured cells were processed in radioimmunoprecipitation assay (RIPA) buffer, 20 µg of each lysate was subjected to electrophoresis using TGX 5-15% midi gel system (BioRad, Hercules, CA, USA) and wet tank transfer to nitrocellulose membrane, as described [62]. After transfer, the membranes were fully dried, rehydrated in nano-pure water, blocked for 30 min with 5% nonfat milk (Bio-Rad 170-6404) or 5% bovine serum albumin (A-9647 Sigma-Aldrich Corp., Saint Louis, MO, USA) in Tris buffered saline (TBS, pH 7.4) with 0.1% Tween 20 (TBS-T) at room temperature. Antibodies were diluted into fresh blocking buffer according to the manufacturer’s recommendations, and the membranes processed as described [62]. Antibodies used were: Cyclin L1 (A302-058A) from Bethyl Laboratories (Montgomery, TX, USA); Cyclin L2 (600-401-878) from Rockland Immunochemicals (Pottstown, PA, USA); Actin (sc-1616) from Santa Cruz Biotechnology (Santa Cruz, CA, USA); CDK11 (5524), MCL-1 (5453), Cyclin A2 (4656), Cyclin B1 (12231), Cyclin D1 (2978), Histone H3 Phospho-Ser10 (3377) from Cell Signaling (Danvers, MA, USA); Survivin (AF886) from R&D Systems (Minneapolis, MN, USA). Proteins were detected by enhanced chemiluminescence using Pierce SuperSignal West Pico and Dura substrates (34078, 34076, Pierce Biotechnology, Rockford, IL, USA). The majority of chemiluminescent signal detection was performed using the Odyssey Fc instrument (LI-COR Biotechnology, Lincoln, NE, USA); however, some signal was captured using Blue Lite autorad film (F-9024-8910, ISC BioExpress, Kaysville, UT, USA). Quantitation of LI-COR signals was performed using Image Studio 5.2 (LI-COR Biotechnology). Quantitation of signal from film images was performed using Image J (National Institutes of Health, Bethesda, MD, USA).

### 4.7. Quantitative Real-Time RT-PCR Analysis

Total RNA was isolated from frozen cell pellets using the RNeasy mini kit (Qiagen, Germantown, MD, USA), including the on-column DNase digestion according to the manufacturer’s protocol, and quantitated using a NanoDrop spectrophotometer (Thermo Fisher Scientific, Waltham, MA, USA). The High-capacity cDNA Reverse Transcription Kit (Thermo Fisher Scientific) was used to synthesize cDNA from total RNA (1.5 µg) using oligo-dT primers according to the manufacturer’s protocol and with the following conditions: 10 min 25 °C; 120 min 37 °C, 5 min 85 °C. Pre-designed primers were obtained from Integrated DNA Technologies (Coralville, IA, USA), Assay Ids: CDK11A Hs.PT.58.3231104; CDK11B Hs.PT.58.28195041; Cyclin A Hs.PT.56a.4535284; Cyclin B Hs.PT.58.39564933; Cyclin D1 Hs.PT.56a.4930170; Cyclin E2 Hs.PT.58.142770; MYC Hs.PT.58.26770695; RPLP0 Hs.PT.58.20222060; SFN Hs.PT.58.20789121; BRCA1 Hs.PT.56a.27724517; WEE1 Hs.PT.58.15526271; TBP Hs.PT.58v.39858774. Reactions were run according to manufacturer’s specification using 96 well FAST plates on a 7900HT machine (Applied Biosystems, Foster City, CA, USA). Analyses were performed using the SDS 2.3 ABI software and changes calculated according to the 2^(−∆∆Ct)^ method. RPLP0 and TBP were used as the reference gene for normalization for Table 2, whereas RPLP0 alone was used for Table 4. All results are reported as the average of reactions run in duplicate.

### 4.8. FACS Analysis of Cultured Cells

A375 and WM1366 cells were transfected in 60 mm plates as described for the Clonal Survival Assays. Forty-eight h post-transfection, cells were collected within the media using a cell lifter and centrifuged 500× g for 5 min at room temperature. Cell pellets were resuspended in 10 mL of phosphate buffered saline and centrifuged 500× g for 5 min at room temperature. Cell pellets were resuspended to 4 × 10^6^ cells per mL in phosphate buffered saline, and 0.5 mL transferred to a 14 mL polypropylene tube. 4.5 mL of ice cold 70% ethanol was added to the cells in a drop-wise manner over a 1 min period and stored at −20 °C for up to 2 weeks. Cells were centrifuged 500× g for 5 min at 4 °C. The supernatant was removed and the cells washed 2 consecutive times in 5 mL of phosphate buffered saline with the same centrifugation. The supernatant was removed, the cells resuspended in 0.5 mL of FxCycle™ PI/RNase Staining Solution (Thermo Fisher Scientific), incubated for 30 min at room temperature, and filtered prior to analysis on a BD FACSAria III (BD Biosciences, San Jose, CA, USA).

### 4.9. NanoString nCounter Expression and Bioinformatics Analysis

Total cellular RNA was isolated from siRNA transfected A375 cells, three samples of siCDK11 and three samples of siControl, as described for q-RT-PCR. The PanCancer Signaling panel was obtained with the standard code set plus these additional genes: CSNK2A1; CSNK2A2; CSNK2B; CDK11A; CDK11B; CCNL1; CCNL2; MCL1; STAT5A; STAT5B. Sample hybridization, purification, immobilization and imaging were performed according to the manufacturer’s protocol (NanoString Technologies). Digital analyzer output reporter code count (RCC) files were analyzed using two R software packages: the NanoStringQCPro package to assess quality and to obtain a normalized data matrix for visualization purposes [63] and the NanoStringDiff package for differential gene expression testing [64]. The raw count data was background subtracted, base 2 log transformed, centered so each gene had a similar mean or median, and scaled so each gene has a similar standard deviation. Sample group comparisons between siCDK11 and siControl were made using the general linear model method employed in the NanoStringDiff R package. The model incorporates useful normalization parameters derived from positive controls, negative controls, and 40 housekeeper genes that can control for lane-to-lane variation, background noise, and RNA input levels, respectively. The list of significantly differentially expressed genes is provided in Table S1. Results are provided including the log-fold change, likelihood ratio value for the comparison, the *p*-value as the unadjusted probability that the gene is not more/less regulated in the first group compared to the second group, and the *q*-value which is a False Discovery Rate (FDR) adjusted *p*-value by the Benjamini and Hochberg method for multiple testing [65]. A *q*-value cutoff value of 0.05 or less and a log2 fold change greater or less than 0.5 was chosen to be included in the “significant” genes list. The full set of data is available in the Gene Expression Omnibus database (GSE128693). Reactome pathway enrichment analysis was performed using a hypergeomectric test implemented in the clusterProfiler R package [66]. Genes significantly up or down-regulated after CDK11 knockdown were compared against multiple Reactome pathway gene lists. Pathways with a Benjamini and Hochberg adjusted *p*-value less than 0.05 were reported.

### 4.10. Human Data Survival Analysis

Survival analysis based on the fragments per kilobase of transcript per million mapped reads (FPKM) value of each gene was performed as described on Human Protein Atlas website (www.proteinatlas.org; Version 18.1; Ensembl version: 88.38). Genes with log rank *p*-values less than 0.001 in maximally separated Kaplan-Meier analysis were defined as prognostic genes. If the group of patients with high expression of a selected prognostic gene has a higher observed event than expected event, it is an unfavourable prognostic gene. Data can be found at the following website: https://www.proteinatlas.org/ENSG00000008128-CDK11A/pathology/tissue/melanoma.

### 4.11. Statistical Analysis

Cell viabilities (ratio of siCDK11 to siNT) were compared to 1 using one sample, two-sided *t*-tests. The number of colonies or tumorsphere areas were compared between matched siCDK11 and siNT data using paired *t*-tests. When sample sizes were at least 3, mean protein levels (ratio of siCDK11 to siNT) were compared to 1 using two-sample one-sided *t*-tests; otherwise means and 95% confidence intervals were presented. Mean percentages of cells in each cell cycle phase were compared between siCDK11 and siNT using two-sample two-sided *t*-tests assuming unequal variances. Data were summarized using Microsoft Excel (Microsoft Corp., Redmond, WA, USA) and analyzed using SAS 9.4 (SAS, Cary, NC, USA).

## Figures and Tables

**Table 1:** Characteristics of melanoma and melanocyte cell lines.

Cell Lines	Transformed	BRAF Mutation	NRAS Mutation
Hema-Lp ^1^	No	None	None
A375	Yes	V600E	None
1205Lu	Yes	V600E	None
WM39	Yes	V600E	None
SK-Mel-2	Yes	None	Q61R
WM1366	Yes	None	Q61L
WM3000	Yes	None	Q61R

^1^ Primary human epidermal melanocytes isolated from adult lightly pigmented skin.

**Table 2:** mRNA expression levels in melanoma cell lines relative to non-transformed melanocytes.

Cell Line	CDK11A	CDK11B	MYC
Hema-Lp	1	1	1
A375	0.06	0.97	3.24
1205Lu	0.28	0.18	1.01
WM39	1.51	0.94	1.68
WM1366	0.10	0.16	1.51
WM3000	0.07	0.40	0.86

mRNA expression normalized to geometric mean of TBP and RPLP0, calculated by delta-delta-Ct method, and expressed relative to Hema-Lp.

**Table 3:** Protein expression levels following siCDK11 transfection in certain melanoma pathways.

Cell Line	Time Point (h)	CDK11	rpS6 P-S235/236	rpS6	Erk 1/2 P-TY	Erk 1/2	β-Catenin Active	β-Catenin	cMYC	MCL-1	Survivin
**A375**	48	0.22	0.89	1.11	1.94	0.87	1.24	1.37	0.90	ND	ND
		(0.09, 0.34)	(0.83, 0.96)	(0.81, 1.40)	(1.17, 2.71)	(0.72, 1.01)	(0.68, 1.81)	(0.78, 1.95)	(0.83, 0.96)		
	72	0.39	0.92	1.27	1.63	ND	ND	1.62	0.18	0.98	0.69
		(0.35, 0.43)	(0.67, 1.17)	0.80, 1.74)	(1.25, 2.02)			(1.21, 2.02)	(0.09, 0.26)	(0.74, 1.22)	(0.55, 0.84)
**WM-1366**	48	0.27	1.28	0.88	0.90	0.92	1.13	0.89	0.36	ND	ND
		(0.15, 0.39)	(0.72, 1.84)	(0.77, 0.99)	(0.72, 1.08)	(0.77, 1.08)	(0.70, 1.56)	(0.65, 1.14)	(0.13, 0.60)		
	72	0.19	1.17	0.77	1.53	0.93	0.68	0.81	0.33	0.67	2.08
		(−0.01, 0.39)	(−0.68, 4.82)	(0.35, 1.19)	(0.84, 2.22)	(0.76, 1.11)	(0.31, 1.06)	(0.27, 1.35)	(0.21, 0.44)	(0.44, 0.91)	(1.79, 2.37)

All values normalized to actin expression and expressed relative to siControl treated cells. Means and 95% confidence intervals presented due to small number of samples. ND = not determined.

**Table 4:** Gene expression changes found by NanoString PanCancer array and q-RT-PCR.

	NanoString: A375		q-RT-PCR: A375	q-RT-PCR: WM1366
Gene	Fold Change ^1^	*q*-Value	Fold Change (95% CI) ^2^	Fold Change (95% CI) ^2^
CCNA2	0.53	1.35 × 10^−9^	0.55 (0.48, 0.62)	1.08 (0.42, 1.75)
CCNB1	0.52	2.72 × 10^−6^	0.38 (0.34, 0.41)	0.91 (0.53, 1.28)
CCND1	0.56	3.65 × 10^−5^	0.49 (0.41, 0.58)	0.37 (0.37, 0.38)
CCNE2	0.66	1.15 × 10^−3^	0.72 (0.66, 0.77)	1.13 (0.20, 1.22)
WEE1	1.70	5.39 × 10^−3^	1.97 (1.44, 2.51)	1.56 (0.72, 2.41)
SFN	0.19	1.95 × 10^−2^	0.25 (0.24, 0.26)	1.10 (0.95, 1.25)
BRCA1	0.51	5.19 × 10^−10^	0.40 (0.31, 0.49)	0.72 (0.51, 0.93)
MYC	1.72	1.45 × 10^−3^	1.40 (1.34, 1.46)	0.71 (0.20, 1.22)

^1^ Mean standard fold change in siCDK11 cells relative to siControl cells based on 3 samples. ^2^ Mean standard fold change in siCDK11 cells relative to siControl cells based on 2 samples; 95% confidence interval presented.

**Table 5:** Protein expression levels following siCDK11 transfection—Cell cycle pathways.

Cell Line	Time (h)	Cyclin A2	Cyclin B1	Cyclin D1	Histone H3 P-S10
A375	48	0.27	0.45	0.72	1.57
		(0.15, 0.39)	(0.27, 0.64)	(0.66, 0.79)	(1.50, 1.65)
	72	ND	ND	ND	2.75 (1.72, 3.79)
WM1366	48	1.14	1.35	0.32	1.99
		(0.48, 1.81)	(0.43, 2.28)	(0.12, 0.52)	(0.59, 3.39)
	72	3.48	2.02	0.51	ND
		(2.80, 4.16)	(1.03, 3.01)	(0.11, 0.91)	

All values normalized to actin expression and expressed relative to siControl treated cells. Means and 95% confidence intervals presented. ND = not determined.

**Table 6:** Effects of siCDK11 on cell cycle distribution at 48 h post-transfection.

Cell Line	Treatment	G1	S	G2/M
A375	Untreated	62.6 ± 1.4	24.7 ± 1.0	12.7 ± 0.6
	siCDK11	84.0 ± 0.7 **	11.4 ± 0.5 ***	4.5 ± 0.5 **
	siControl	68.4 ± 0.3	21.1 ± 0.3	10.5 ± 0.3
WM1366	Untreated	56.2 ± 4.3	23.6 ± 2.9	20.3 ± 1.5
	siCDK11	76.6 ± 0.3 *	16.1 ± 0.1 *	7.2 ± 0.5 **
	siControl	56.4 ± 2.7	23.1 ± 2.8	20.5 ± 0.2

The mean ± standard deviation of three experiments is presented. For comparisons between siCDK11 and siControl cells, *** denotes *p*-value < 0.0001; ** *p* < 0.001; * *p* ≤ 0.05.

## Supplementary Materials

The following are available online at https://www.mdpi.com/1424-8247/12/2/50/s1, Table S1: Significantly differentially expressed genes identified in A375 cells using the PanCancer Pathways panel (NanoString Technologies). Table S2: Data from 102 patients included in survival analysis from The Protein Atlas.
