# Supplementary material for: CDK11 Loss Induces Cell Cycle Dysfunction and Death of BRAF and NRAS Melanoma Cells
Source: Pharmaceuticals (Basel). 2019 Apr 2;12(2):50. doi: 10.3390/ph12020050 (PMC6631185; doi:10.3390/ph12020050)
Supplement: Supplementary file 1 [file pharmaceuticals-12-00050-s001.zip › Cover page for Supplemental Tables.docx]

CDK11 Loss Induces Cell Cycle Dysfunction and Death of BRAF and NRAS Melanoma Cells

Rehana L. Ahmed ^1,6^, Daniel P. Shaughnessy ^2^, Todd P. Knutson ^3,4^, Rachel I. Vogel ^5,6^,
Khalil Ahmed ^2,3,6,7^, Betsy T. Kren ^2,6^ and Janeen H. Trembley ^2,3,6,^*

^1^ Department of Dermatology, University of Minnesota, Minneapolis, MN 55455, U.S.A.; [ahme0056@umn.edu](mailto:ahme0056@umn.edu)

^2^ Research Service, Minneapolis VA Health Care System, Minneapolis, MN 55417, U.S.A.; shaug028@umn.edu (D.P.S); ahmedk@umn.edu (K.A.); krenx@umn.edu (B.T.K.);
trem0005@umn.edu (J.H.T.)

^3^ Department of Laboratory Medicine and Pathology, University of Minnesota, Minneapolis, MN 55455, U.S.A.; knut0297@umn.edu (T.P.K.)

^4^ Minnesota Supercomputing Institute, University of Minnesota, Minneapolis, MN 55455, U.S.A.

^5^ Department of Obstetrics, Gynecology and Women’s Health, University of Minnesota, Minneapolis, MN 55455, U.S.A.; isak0023@umn.edu (R.I.V.)

^6^ Masonic Cancer Center, University of Minnesota, Minneapolis, MN 55455, U.S.A.

^7^ Department of Urology, University of Minnesota, Minneapolis, MN 55455, U.S.A.

***** Correspondence: trem0005@umn.edu
