# Supplementary material for: CDK11 Loss Induces Cell Cycle Dysfunction and Death of BRAF and NRAS Melanoma Cells
Source: Pharmaceuticals (Basel). 2019 Apr 2;12(2):50. doi: 10.3390/ph12020050 (PMC6631185; doi:10.3390/ph12020050)
Supplement: Supplementary file 1 [file pharmaceuticals-12-00050-s001.zip › pharmaceuticals-456474 supp/Table S1.pdf]

Table S1. Significantly differentially expressed genes identified in A375 cells using the PanCancer Pathways panel					
Gene	log Fold Change (base 2)	likelihood ratio	p-value	q-value	gene description
DLL4	34.24633383	10.95023505	9.36E-04	0.00814799	delta like canonical Notch ligand 4
RASGRP1	3.849823722	7.758752157	0.00534529	0.0332396	RAS guanyl releasing protein 1
FGF7	2.756868365	30.59894772	3.17E-08	1.17E-06	fibroblast growth factor 7
NR4A3	2.10117785	15.69648452	7.44E-05	9.65E-04	nuclear receptor subfamily 4 group A member 3
DUSP10	1.38589944	48.34190796	3.58E-12	5.30E-10	dual specificity phosphatase 10
MAP3K13	1.356829915	15.73905645	7.27E-05	9.61E-04	mitogen-activated protein kinase kinase kinase 13
BMPR1B	1.165341688	7.735556045	0.00541439	0.03338873	bone morphogenetic protein receptor type 1B
IL24	1.150733409	12.49121538	4.09E-04	0.00403419	interleukin 24
GHR	1.065135437	14.89092593	1.14E-04	0.00138185	growth hormone receptor
SOCS2	1.039055915	13.91198558	1.92E-04	0.00205455	suppressor of cytokine signaling 2
RXRG	1.030917173	11.04686809	8.88E-04	0.00783298	retinoid X receptor gamma
ITGB8	0.991245707	36.02443157	1.95E-09	8.48E-08	integrin subunit beta 8
FOS	0.826687914	19.46308036	1.03E-05	1.85E-04	Fos proto-oncogene, AP-1 transcription factor subunit
MYC	0.789560539	14.74447121	1.23E-04	0.00144603	MYC proto-oncogene, bHLH transcription factor
WEE1	0.762398124	22.1276268	2.55E-06	5.39E-05	WEE1 G2 checkpoint kinase
INHBA	0.720047146	13.94113134	1.89E-04	0.00205269	inhibin beta A subunit
CDK2	0.647563304	19.80569938	8.57E-06	1.63E-04	cyclin dependent kinase 2
NFKBIZ	0.644424945	6.860935426	0.00881004	0.04938963	NFKB inhibitor zeta
NOTCH2	0.634515107	12.66625051	3.72E-04	0.00372315	notch 2
IL6R	0.609554537	9.556363046	0.00199257	0.01445594	interleukin 6 receptor
FBXW7	0.59605282	8.804317878	0.00300518	0.02040217	F-box and WD repeat domain containing 7
IL1RAP	0.566197855	10.18791189	0.00141364	0.01124834	interleukin 1 receptor accessory protein
CTNNB1	0.560456986	12.31465908	4.49E-04	0.00431905	catenin beta 1
PDGFD	0.520849617	9.634166591	0.00190991	0.01403768	platelet derived growth factor D
PCNA	-0.55050283	7.414664734	0.00646944	0.03860793	proliferating cell nuclear antigen
MAP2K1	-0.56841631	17.11296453	3.52E-05	5.32E-04	mitogen-activated protein kinase kinase 1
MDC1	-0.57003873	14.38256345	1.49E-04	0.00169833	mediator of DNA damage checkpoint 1
MAPK1	-0.57141159	22.45482379	2.15E-06	4.68E-05	mitogen-activated protein kinase 1
RFC3	-0.57807524	18.17711238	2.01E-05	3.24E-04	replication factor C subunit 3

Gene	log Fold Change (base 2)	likelihood ratio	p-value	q-value	gene description
MSH6	-0.5894768	23.40937061	1.31E-06	3.23E-05	mutS homolog 6
FANCA	-0.59550683	7.988122421	0.00470852	0.03056409	Fanconi anemia complementation group A
CCNE2	-0.60124371	15.29965503	9.17E-05	0.00115055	cyclin E2
UBE2T	-0.6087933	22.69099517	1.90E-06	4.27E-05	ubiquitin conjugating enzyme E2 T
TTK	-0.61180397	19.15140981	1.21E-05	2.13E-04	TTK protein kinase
CDC25B	-0.61361832	25.34162618	4.80E-07	1.37E-05	cell division cycle 25B
PPARG	-0.61769858	24.26217	8.41E-07	2.22E-05	peroxisome proliferator activated receptor gamma
TIAM1	-0.6178792	8.89190518	0.00286438	0.01962629	T-cell lymphoma invasion and metastasis 1
FANCC	-0.61842668	10.85014665	9.88E-04	0.00850036	Fanconi anemia complementation group C
BRIP1	-0.62908169	16.76201189	4.24E-05	6.05E-04	BRCA1 interacting protein C-terminal helicase 1
MAP2K6	-0.64321301	16.23148333	5.61E-05	7.68E-04	mitogen-activated protein kinase kinase 6
CDC7	-0.65031292	14.49905056	1.40E-04	0.00162141	cell division cycle 7
CHEK1	-0.65771479	28.6264528	8.78E-08	2.95E-06	checkpoint kinase 1
PKMYT1	-0.66257577	7.577652666	0.00590963	0.0355539	protein kinase, membrane associated tyrosine/threonine 1
BAD	-0.67145767	14.79547137	1.20E-04	0.00143014	BCL2 associated agonist of cell death
MSH2	-0.67961208	31.35289475	2.15E-08	8.38E-07	mutS homolog 2
XRCC4	-0.68250984	20.78169099	5.15E-06	1.05E-04	X-ray repair cross complementing 4
FGFR1	-0.68751608	9.628362224	0.00191595	0.01403768	fibroblast growth factor receptor 1
VEGFC	-0.69897605	9.460364469	0.00209959	0.01508446	vascular endothelial growth factor C
BRCA2	-0.70382538	19.62777418	9.41E-06	1.74E-04	BRCA2, DNA repair associated
HELLS	-0.70873208	18.83318926	1.43E-05	2.46E-04	helicase, lymphoid specific
EPHA2	-0.70961324	18.66647243	1.56E-05	2.62E-04	EPH receptor A2
CDKN2C	-0.72609421	24.33047773	8.11E-07	2.22E-05	cyclin dependent kinase inhibitor 2C
SETBP1	-0.73914232	9.313583175	0.00227461	0.0160306	SET binding protein 1
MCM7	-0.74123211	36.64959364	1.41E-09	6.98E-08	minichromosome maintenance complex component 7
FANCB	-0.75048102	18.50789381	1.69E-05	2.78E-04	Fanconi anemia complementation group B
WHSC1	-0.76549798	16.97802475	3.78E-05	5.60E-04	NA
SMAD3	-0.76990233	17.18314506	3.39E-05	5.23E-04	SMAD family member 3
POLE2	-0.77672428	13.07297075	3.00E-04	0.00312246	DNA polymerase epsilon 2, accessory subunit
CDK11A	-0.78511625	17.68958239	2.60E-05	4.09E-04	cyclin dependent kinase 11A

Gene	log Fold Change (base 2)	likelihood ratio	p-value	q-value	gene description
IGFBP3	-0.80064979	7.362621	0.00665936	0.03942344	insulin like growth factor binding protein 3
PRKCA	-0.80083789	16.71925551	4.33E-05	6.05E-04	protein kinase C alpha
ITGA3	-0.828689	14.08578633	1.75E-04	0.00195829	integrin subunit alpha 3
CCND1	-0.84027787	23.11364013	1.53E-06	3.65E-05	cyclin D1
MAD2L2	-0.85747819	42.64049496	6.58E-11	4.12E-09	mitotic arrest deficient 2 like 2
IL8	-0.87365117	36.37423469	1.63E-09	7.53E-08	NA
FGFR3	-0.88130128	7.092751072	0.00773963	0.04439791	fibroblast growth factor receptor 3
LIF	-0.8934768	12.7301624	3.60E-04	0.00364734	LIF, interleukin 6 family cytokine
FANCG	-0.89387	26.08404278	3.27E-07	9.68E-06	Fanconi anemia complementation group G
HIST1H3H	-0.90327207	27.65258629	1.45E-07	4.52E-06	histone cluster 1 H3 family member h
CCNA2	-0.90692624	45.1542801	1.82E-11	1.35E-09	cyclin A2
RFC4	-0.90890672	23.94897712	9.89E-07	2.52E-05	replication factor C subunit 4
STMN1	-0.92292817	51.66328666	6.59E-13	2.44E-10	stathmin 1
MCM2	-0.92552278	15.9687372	6.44E-05	8.66E-04	minichromosome maintenance complex component 2
ETV4	-0.94645047	27.6323298	1.47E-07	4.52E-06	ETS variant 4
CCNB1	-0.95061856	28.87650682	7.71E-08	2.72E-06	cyclin B1
BRCA1	-0.97696923	48.82105216	2.80E-12	5.19E-10	BRCA1, DNA repair associated
C19orf40	-0.98283476	34.57268323	4.11E-09	1.69E-07	NA
GNAQ	-0.98578856	42.6085488	6.69E-11	4.12E-09	G protein subunit alpha q
HIST1H3B	-1.00673311	45.80180177	1.31E-11	1.35E-09	histone cluster 1 H3 family member b
MCM5	-1.03326006	20.74087765	5.26E-06	1.05E-04	minichromosome maintenance complex component 5
HIST1H3G	-1.07545582	22.85902616	1.74E-06	4.03E-05	histone cluster 1 H3 family member g
WNT5A	-1.13054321	42.30044402	7.83E-11	4.46E-09	Wnt family member 5A
FN1	-1.13297179	45.41178742	1.60E-11	1.35E-09	fibronectin 1
HSPA1A	-1.14438845	10.65088406	0.00110018	0.00914759	heat shock protein family A (Hsp70) member 1A
E2F1	-1.18035789	38.13075081	6.62E-10	3.50E-08	E2F transcription factor 1
DDB2	-1.25067164	48.9368901	2.64E-12	5.19E-10	damage specific DNA binding protein 2
BCL2L1	-1.27793873	45.26361911	1.72E-11	1.35E-09	BCL2 like 1
PIM1	-1.3619312	10.12740911	0.00146081	0.01143576	Pim-1 proto-oncogene, serine/threonine kinase
MGMT	-1.49249033	81.41095568	0	0	O-6-methylguanine-DNA methyltransferase

Gene	log Fold Change (base 2)	likelihood ratio	p-value	q-value	gene description
RASAL1	-1.59998716	7.858121127	0.00505929	0.03172778	RAS protein activator like 1
IRS1	-2.1241589	45.88028847	1.26E-11	1.35E-09	insulin receptor substrate 1
CDK11B	-2.35297023	13.99755866	1.83E-04	0.00202173	cyclin dependent kinase 11B
BMP4	-2.36333979	16.72101306	4.33E-05	6.05E-04	bone morphogenetic protein 4
SFN	-2.3959464	8.921733875	0.00281797	0.01948875	stratifin
LFNG	-2.49561756	15.22695516	9.53E-05	0.00117577	LFNG O-fucosylpeptide 3-beta-N-acetylglucosaminyltransferase
PLA2G3	-3.68836457	10.11822322	0.0014681	0.01143576	phospholipase A2 group III
ITGB4	-4.09165151	19.99024789	7.78E-06	1.52E-04	integrin subunit beta 4
