# Supplementary material for: CDK11 Loss Induces Cell Cycle Dysfunction and Death of BRAF and NRAS Melanoma Cells
Source: Pharmaceuticals (Basel). 2019 Apr 2;12(2):50. doi: 10.3390/ph12020050 (PMC6631185; doi:10.3390/ph12020050)
Supplement: Supplementary file 1 [file pharmaceuticals-12-00050-s001.zip › pharmaceuticals-456474 supp/Table S2 v2.pdf]

**Table S2. Data from 102 patients included in survival analysis from The Human Protein Atlas**

| | Age in years Mean (range) | n (%) | Stage | n (%) | Primary Tumor Location |
|---|---|---|---|---|---|
| Data Missing (n = 29) | | | | | |
| Male (n = 43) | 60.7 (24-90) | 0 | I | 6 (14.0) | Head and neck |
| | | 22 (51.2) | II | 12 (27.9) | Extremities |
| | | 13 (30.2) | III | 21 (48.8) | Trunk |
| | | 2 (4.6) | IV | 4 (9.3) | Other/unknown |
| | | 6 (14.0) | unknown | | |
| Female (n = 30) | 65.7 (41-84) | 1 (3.3) | I | 2 (6.7) | Head and neck |
| | | 15(50.0) | II | 17 (56.7) | Extremities |
| | | 10 (33.3) | III | 11 (36.6) | Trunk |
| | | 1 (3.3) | IV | 0 | Other/unknown |
| | | 3 (10.0) | unknown | | |

Race: 3 male and 1 female were Asian. All other patients for whom we have data were White.
